# The Influence of Apparent Temperature on Mortality in the Kintampo Health and Demographic Surveillance Area in the Middle Belt of Ghana: A Retrospective Time-Series Analysis

**DOI:** 10.1155/2020/5980313

**Published:** 2020-09-22

**Authors:** Kenneth Wiru, Felix Boakye Oppong, Oscar Agyei, Charles Zandoh, Obed Ernest Nettey, Robert Adda, Antonio Gasparrini, Kwaku Poku Asante

**Affiliations:** ^1^Kintampo Health Research Centre, Ghana Health Service, Bono East Region, Kintampo, Ghana; ^2^Department of Social and Environmental Health Research, Faculty of Public Health and Policy, London School of Hygiene and Tropical Medicine, London, UK

## Abstract

Globally, studies have shown that diurnal changes in weather conditions and extreme weather events have a profound effect on mortality. Here, we assessed the effect of apparent temperature on all-cause mortality and the modifying effect of sex on the apparent temperature-mortality relationship using mortality and weather data archived over an eleven-year period. An overdispersed Poisson regression and distributed lag nonlinear models were used for this analysis. With these models, we analysed the relative risk of mortality at different temperature values over a 10-day lag period. By and large, we observed a nonlinear association between mean daily apparent temperature and all-cause mortality. An assessment of different temperature values over a 10-day lag period showed an increased risk of death at the lowest apparent temperature (18°C) from lag 2 to 4 with the highest relative risk of mortality (RR = 1.61, 95% CI: 1.2, 2.15, *p* value = 0.001) occurring three days after exposure. The relative risk of death also varied between males (RR = 0.31, 95% CI: 0.10, 0.94) and females (RR = 4.88, 95% CI: 1.40, 16.99) by apparent temperature and lag. On the whole, males are sensitive to both temperature extremes whilst females are more vulnerable to low temperature-related mortality. Accordingly, our findings could inform efforts at reducing temperature-related mortality in this context and other settings with similar environmental and demographic characteristics.

## 1. Introduction

The effects of changing weather conditions and their extremes on human health have gained much currency in environmental epidemiology. Vagaries of temperature, in particular, have been reported to significantly affect mortality [1, 2]. Till date, this phenomenon represents a lethal threat to human survival especially in an era where global ambient temperatures are fast increasing largely due to anthropogenic activities [3].

Evidence suggests that the weather-mortality relationship varies among geographic regions owing to differences in climatic conditions, which influence the severity and duration of exposure to weather events. Moreover, it has been posited that, besides variations in regional climatic conditions, spatial variations in the weather-mortality association could result from differences in sociodemographic and economic characteristics of populations and the level of urbanity of communities which modify the severity of exposure to cold or heat and adaptation mechanisms [4–6]. Population subgroups such as the aged, young children, and persons with cardiovascular and respiratory conditions are more vulnerable to temperature-related mortality due to their poor physiological ability to adjust to cold or heat exposures [1, 4, 6]. Furthermore, it is evident that urban residents may be exposed to higher ambient temperatures than their rural and suburban counterparts due to the “urban heat island effect” [1]. Hence, the risk of heat-related mortality could be higher in urban environments with persistently high day and night temperatures.

The effect of variable weather on human health has been greatly explored in developed countries [7, 8], providing relevant information for the development of weather warning systems to safeguard people against adverse consequences of extremely hot or cold events including death. More recent evidence points to a significant association between hot and cold temperature extremes and preterm births in the United States [9] whilst rises in daily mean temperature beyond specific thresholds have been reported to substantially increase the risk of all-cause mortality with significant variations across space and among population subgroups in the United Kingdom and Australia, [10]. Likewise, Borg and colleagues [11] have documented a significant association between daily rises in temperature and the incidence of renal diseases, whereas Huang et al. [12] established an association between cold and hot temperature thresholds and cardiovascular disease mortality in a subtropical environment in China. Additionally, Ou et al. reported a considerable influence of low relative humidity on mortality attributable to cardiovascular and ischemic heart diseases in a similar environment [13].

Though the influence of weather on population health has been underexplored in developing countries, particularly in sub-Saharan Africa, findings from a few Health and Demographic Surveillance sites in Africa largely parallel those characterized in other regions. In Kenya, for example, Egondi et al. found high temperatures to effectively influence child mortality and deaths due to noncommunicable diseases [14]. Besides, Mrema and colleagues described a strong association between weather elements and under-five mortality in Tanzania, whereas, in Burkina Faso and Ghana, Diboulo et al. and Azongo and colleagues, respectively, documented significant effects of climatic factors on mortality with varied effect estimates among population subgroups [15–17].

Ghana experiences a tropical climate, characterized by seasonal and spatial vagaries of weather episodes with adverse implications for population health. Besides, the World Health Organization (WHO) projects that heat-related mortality among the Ghanaian elderly (aged 65 years and above) could rise to about 70 deaths per 100,000 population by the year 2080 owing to the endless upsurge in atmospheric temperature [18]. Considering this caveat and the spatial character of climatic conditions in the country, a region-specific analysis of the weather–mortality relationship is indispensable. Such an analysis promises to contribute relevant information to the design of local- and country-scale public health interventions targeted at mitigating weather-related mortality.

We aimed to explore the effect of apparent temperature (AT)-(a composite measure of ambient temperature and relative humidity) on all-cause mortality as well as the modifying effect of sex on the apparent temperature-mortality association in the Kintampo Health and Demographic Surveillance area of Ghana's middle belt. Given the joint effect of ambient temperature and relative humidity on human comfort, we used AT as our exposure variable since it takes into consideration the influence of humidity, which makes it a better indicator of human heat exposure than ambient temperature alone [19]. Our aim was ultimately achieved using time-series weather and mortality data collected from 2005 to 2015.

## 2. Methods

### 2.1. Study Area

The Kintampo Health and Demographic Surveillance area is located in the Bono East region of Ghana's middle belt. It covers two contiguous districts, the Kintampo North Municipality and South district, both of which lie between longitudes 1°20′ and 2°10′ west and latitudes 8°45′ and 7°45′ north. Westwards, it is bounded by the Banda and Bole districts, whereas it is bounded to the east by the Pru and East Gonja districts. It is also bounded to the north by the Black Volta River and to the south by the Wenchi Municipality, Techiman North, and Nkoranza North districts. The area comprises of 161 communities covering a surface area of about 6,621 square kilometers. It is largely characterized by a forest-savannah mix of vegetation as it is the transitional zone between the southern forest and northern savannah regions of the country [20]. The area has a tropical continental type of climate and experiences a double maxima rainfall regime (major and minor rainy seasons) with mean annual rainfall being between 1400 and 1800 millimeters. Surface air temperature and humidity also assume a seasonal character with low mean monthly temperature ranging between 24°C in August and 30°C in March. Humidity is generally high ranging between 70% and 100% for the most part of the year, whereas relatively low amounts (<50%) are recorded between December and January. Much of the area is rural with peasant farming being the mainstay of majority of the inhabitants.

**Figure 1:**
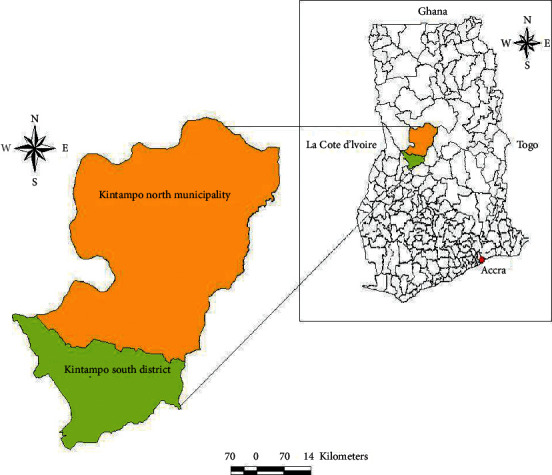
Map of Ghana highlighting the Kintampo Health and Demographic Surveillance area.

The study area has a population of 156,145 [21], which is longitudinally tracked for updates on births, deaths, in- and out-migrations, pregnancies, education, socioeconomic status, prevalence of common illnesses, specific causes of death, and information on other health and demographic variables of public health significance. The area is ethnically diverse with the Mo and Bono people being the indigenous ethnic groups. Moreover, it is settled by many immigrant tribes of northern descent, Gas, Ewes, Dangbes, and other Akan tribes [20]. There are several formal health facilities from which people access health services in the study area. Two of these are general hospitals, respectively, located in the Kintampo and Jema townships with the rest of them being six public health centres, thirty-one Community-Based Health Planning and Services (CHPS) Compounds, four private clinics, and two private maternity homes.  Figure 1 is a map of the study area.

### 2.2. Mortality Data

We used daily counts of all-cause mortality recorded by the KHDSS between 2005 and 2015 for this analysis. These data were collected by trained fieldworkers who routinely visited all households within the study area in well-defined cycles for updates on vital health and demographic events that occurred after the last visit by administering specially designed questionnaires to household heads or knowledgeable adult household members. Community Key Informants (CKIs) complemented the work of fieldworkers by recording and forwarding events in their respective communities to the HDSS office through a designated field supervisor in order to ensure a complete record of events or minimalize missing data. Trained field supervisors and research assistants conducted quality checks in order to enhance the integrity of the data. Death heaping on specific dates of the month was sorted using the method described by Azongo and colleagues [17].

### 2.3. Weather Data

We obtained time-series weather data (daily minimum and maximum temperatures and relative humidity) spanning the analysis period (2005–2015) from the Ghana Meteorological Agency and the United States' National Oceanic and Atmospheric Administration's (NOAA) National Climate Data Centre. Missing data from the data obtained from the Ghana Meteorological Agency were imputed with data from NOAA's National Climate Data Centre. A composite measure (daily mean apparent temperature) was computed from the daily minimum and maximum temperature and relative humidity data. Apparent temperature encompasses the combined effect of air temperature and relative humidity. This measure of temperature has been used as an exposure index in several previous studies [19, 22, 23]. Apparent temperature was computed using the formula by Schoen, 2005 [24]:

(1) $$\begin{array}{c} \text{Apparent temperature}=\text{T}-1.0799\times {\text{e}}^{0.03755\times \text{T}}\left[1-{\text{e}}^{0.0801\times \left(\text{D}-14\right)}\right], \end{array}$$

T is temperature in degree Celsius (°C) and D is dew point temperature (°C) computed as follows:

(2) $$\begin{array}{c} \text{D}=\frac{237\text{.}3\times \left(\left(17\text{.}27\mathrm{\times T}/\mathrm{237}\mathrm{.3+T}\right)+\ln\left(\text{RH}\right)\right)}{17\text{.}27-\left(\left(17\text{.}27\mathrm{\times T}/\mathrm{237}\mathrm{.3+T}\right)+\ln\left(\text{RH}\right)\right)}, \end{array}$$

where *RH*is the relative humidity.

Though this indicator encompasses the effect of relative humidity, prior analysis has shown that different measures of temperature generally have a comparable effect on mortality [25].

### 2.4. Statistical Analysis

An overdispersed Poisson regression model together with a distributed lag nonlinear model (DLNM) [26, 27] was used to examine the short-term association of daily mean apparent temperature (AT) with all-cause mortality. The overdispersed Poisson regression model has been used in many studies to assess the weather–mortality association [28–31] and to account for Poisson overdispersion [28]. With the DLNM, the nonlinear effects of temperature, humidity, and lag were modeled simultaneously. DLNM is suitable for assessing the temperature-mortality association which often shows a J-, W-, V-, or U-shaped relationship [28, 29]. A natural cubic spline DLNM was used for the nonlinear effect of both apparent temperature and lag. A 10-day lag period was used to capture the delayed effect of apparent temperature. The apparent temperature with overall minimum mortality (24.8°C) was defined as the reference value for calculating the relative risk of mortality. AIC for quasi-Poisson models (Q-AIC) was used to select the degrees of freedom (df) for AT and lag [28, 32]. The final model employed a natural cubic spline of apparent temperature with 4 df and a natural cubic spline with 3 df for lag days. For controlling for trends and seasonal patterns in mortality, a natural cubic spline with 7 df per year was used [26, 29]. Also, we included a categorical variable for day of the week as a control variable.

We derived estimates of the overall cumulative relative risk, measuring the net effect across the whole lag period, and also lag-specific contributions. These, we also summarized graphically by reporting the overall cumulative exposure-response curve and the lag-response curves at various temperature values. The relative risks of low, first quartile, third quartile, and high apparent temperatures were calculated by comparing with the reference value. Additionally, a stratified analysis by sex was performed.

For all statistical tests, relative risks and 95% confidence intervals were reported. Data analysis was done using Stata version 14.0 and *R* version 3.4.3. Stata was used for the data preparation whilst *R* was used for the DLNM using the “splines” and “dlnm” package [33].

## 3. Results

**Figure 2:**
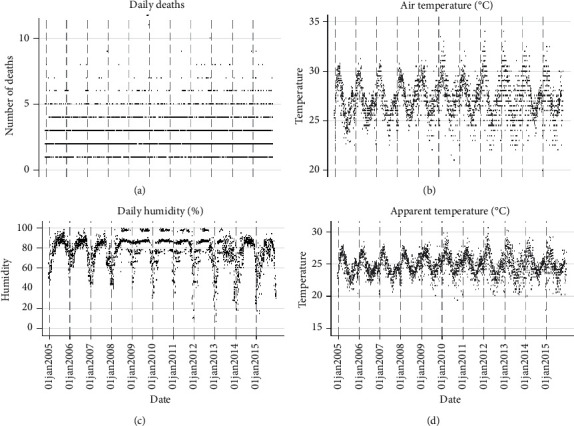
Time series of daily mortality, mean temperature, and humidity in the Kintampo HDSS area from 2005 to 2015.

A total of 10,865 all-cause deaths were recorded in the Kintampo Health and Demographic Surveillance area during the study period (2005–2015) (Table 1). There was a daily mean mortality of 2.7 (SD = 1.3, range 1–11) in the population. Significantly more males than females died over the analysis period (56.6% vs. 43.4%; *p* value < 0.001). The highest mortality count was observed among the 5–19-year age group (35.7%) followed by persons 60 years and older. There were slight annual variations in death counts despite the absence of a clear-cut downward or upward trend over the study period (Figure 2).

Seasonal fluctuations in temperature were also observed with relatively low temperatures occurring at the start of the dry season (between November and December). However, temperatures were generally high between January and April with moderately low temperatures prevailing at the beginning and peak of the rainy season (between May and August) followed by a steady rise in September and October (Figure 2). Relative humidity also assumed a seasonal pattern with lower amounts recorded in the early part of the dry season between November and January when the northeast trade winds were prevalent. On average, the area recorded a daily mean relative humidity of 76.9% whereas the proportions of minimum and maximum daily mean relative humidity were 65.9% and 87.9% apiece (Table 2).

### 3.1. Relationship between Mean Daily Apparent Temperature and Mortality

**Figure 3:**
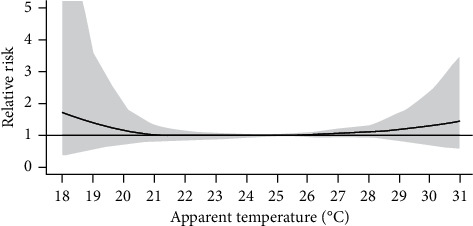
Overall effect of apparent temperature on mortality in the Kintampo HDSS area from 2005 to 2015.

**Figure 4:**
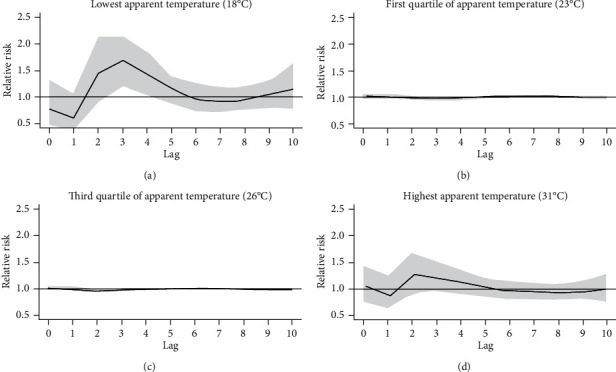
Relative risk of mortality by lag at specific apparent temperatures in the Kintampo HDSS area from 2005 to 2015.

We examined the association between daily mean apparent temperature and all-cause mortality risk in the general population. As shown in Figure 3, there is a nonlinear relationship between mean daily apparent temperature and mortality. Furthermore, we assessed the effects of specific apparent temperatures on mortality risk over a 10-day lag period at the lowest apparent temperature (18°C), first quartile AT (23°C), third quartile AT (26°C), and the highest AT (31°C) (Figure 4). Following this, an increased risk of mortality was observed at the lowest AT from lag 2 to 4. At the lowest AT of 18°C, the highest relative risk of mortality (RR = 1.61, 95% CI: 1.21, 2.15, *p*-value = 0.001) was observed three days after exposure (Figure 4). There was no significant relationship between the first quartile AT, third quartile AT, the highest AT, and daily mortality (Figure 4).

**Figure 5:**
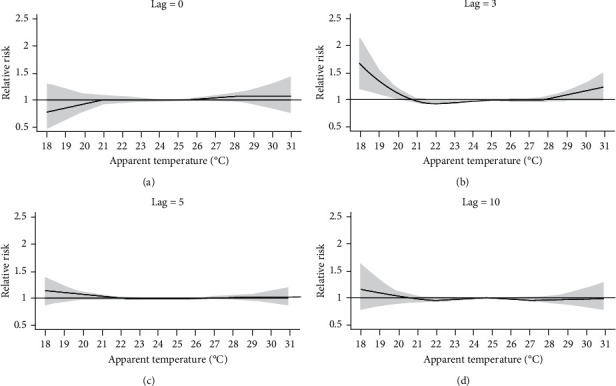
Relative risk of mortality by apparent temperature at specific lags in the Kintampo HDSS area from 2005 to 2015.

Similarly, an exploration of lag-specific effects of AT on mortality showed a higher relative risk at apparent temperatures of 18°C (RR = 1.61, 95% CI: 1.21, 2.15, *p*-value = 0.001) and 19°C (RR = 1.32, 95% CI: 1.10, 1.57, *p*-value = 0.003) three days after exposure. However, there was no significant relationship between AT and mortality for the other specific lags as shown in Figure 5.

### 3.2. Sex-Specific Analysis of the Relationship between Apparent Temperature and Mortality

Further analysis of the modifying effect of sex on the temperature–mortality relationship at different apparent temperatures and lags showed that the relative risk of death was significantly lower (RR = 0.31, 95% CI: 0.10, 0.94) for males after exposure to the lowest apparent temperature (18°C) compared to the reference apparent temperature at lag 0 to 1. However, at the same apparent temperature of 18°C, the relative risk of mortality (RR = 4.88, 95% CI: 1.40, 16.99) was higher for females two to four days after exposure compared to the reference temperature. Also, a considerably higher relative risk of death (RR = 2.19, 95% CI: 1.03, 4.65) was observed for males two to four days after exposure to the highest apparent temperature (31°C). However, there was no significant association between the other specific apparent temperatures (first quartile, third quartile, and the highest AT) and female mortality at the specified lags (Table 3).

## 4. Discussion

This study explored the influence of two climatic factors, temperature and relative humidity (apparent temperature), on all-cause/age mortality in a largely rural population in the middle belt of Ghana over an eleven year period. In general, our analysis revealed a statistically significant evidence of higher risks of mortality at low daily mean AT in the studied population. Precisely, the risk of death was highest at the lowest AT of 18°C. Apparent temperature also showed delayed effects on mortality after our lag strata analysis with the highest risk of death observed at the lowest mean daily AT two to four days after exposure. Though we have not examined specific causes of death in the current analysis, we suppose that the strong effect estimates for low AT observed could emanate from deaths attributable to cardiovascular and respiratory conditions [4, 6], which are commonly associated with low temperatures.

Though previous studies have used a variety of indices in addition to AT which makes direct comparisons complex, we subscribe to the notion that all temperature measures have a comparable effect on mortality [25]. Hence, our finding of a considerable effect of low AT on mortality risk in this study population is consistent with the results of several prior studies on the weather–mortality relationship in other jurisdictions [15, 34–36]. Generally, our finding of a strong effect of low apparent temperature on all-cause mortality in the general population parallels the effect estimates of cold exposures on all-cause and female mortality documented by Dang et al. [34] in Vietnam. Similarly, a population-based study in Abhoynagar, rural Bangladesh, described a strong association between low weekly mean temperature and mortality [35]. The congruence of these findings with our results is probably due to the prevalence of tropical ecological conditions in the respective study settings. However, the lag effects of low apparent temperature observed in the current study contradict the universal notion that cold weather episodes have a gradual and enduring effect on human health as the effect appears to be more immediate (lag 0-1) at a low AT, particularly for the male population, than the effect of high AT which sets in two to four days after exposure.

On the contrary, Azongo and colleagues [17] found no significant association between low temperature and mortality though they reported significant effects of heat in Navrongo, Northern Ghana. One similarity between the findings of our study and that of Navrongo is the observation of a higher risk of male mortality at the highest AT (31°C) which compares with the rise in male mortality at temperatures above 30.06°C in Navrongo [17] though at different lag periods (lag 0-1 for Navrongo and 2–4 for Kintampo). Furthermore, the divergence between findings of the current analysis and those of the Navrongo study is anticipated. This is because local environmental conditions in northern Ghana differ from the conditions prevalent in the middle belt of the country in which the present study was conducted. For instance, northern Ghana lies within the Guinea savannah climatic zone with a single maximum rainfall regime, usually followed by a long dry spell characterized by cold night and hot day temperatures during the harmattan period (November–March). Conversely, the middle belt experiences a double maxima rainfall regime with cold temperatures at the peak of each of the rainy seasons and the harmattan period amidst significant variations in other ecological conditions.

Furthermore, the contrasts between our findings and those of previous studies could stem from differences in sociodemographic and economic characteristics of the studied populations which are known to modify the weather-mortality relationship [5, 6]. Kintampo, for instance, is a predominantly rural agrarian society with most of its inhabitants exposed to cold weather during the rainy and harmattan seasons as they work on their farms. Hence, farming activities and local housing characteristics probably exacerbate people's vulnerability to cold-related mortality as most houses in rural communities are built with wattle, daub, and thatch, which can hardly attract and retain heat during the night. We further believe that the rurality of the study area is a factor that predisposes inhabitants to high mortality risks from cold weather since rural areas are generally known to have low temperatures due to the lack of an “urban heat island effect” [1]. On the contrary, the observed significant effects of high AT (31°C) on all deaths and male-specific mortality could be as a result of long term exposure to heat during the hot season (January–April) and people's inability to afford cooling systems and protective clothing. Besides, our finding that men are susceptible to both low and high apparent temperatures whilst women are more sensitive to low apparent temperature allows for proper targeting of interventions for reducing temperature-related mortality in the study area.

The strength of this study lies in our use of population-level mortality data which was continuously updated throughout the analysis period. However, a major weakness is our inability to stratify the analysis by age, largely due to low mortality numbers for the respective population age groups. An attempt at such stratified analysis produced results with unreasonably wide confidence intervals, which we deemed unreliable. Hence, we were unable to assess the modifying effect of age on apparent temperature-related mortality in the study area, which could have provided more information on the strategic targeting of interventions towards mitigating temperature-related mortality.

## 5. Conclusions

This study revealed the influence of apparent temperature on mortality in the rural middle belt of Ghana. The core findings demonstrate that the population of the Kintampo area is vulnerable to both low and high apparent temperatures with the male population being susceptible to both temperature extremes whilst females are more sensitive to low apparent temperature. We have demonstrated that the effect of low apparent temperature on male mortality is faster than its effect on female deaths though both low and high apparent temperatures appear to impact all deaths at the same lag period. Consequently, our findings hold potential to inform local and country-scale interventions aimed at attenuating temperature-related mortality, especially in this era of global warming with untold public health ramifications.

## Figures and Tables

**Table 1:** Summary statistics of daily mortality in the Kintampo HDSS area from 2005 to 2015.

Classification	Total mortality	Daily mean (SD)	Min-max	Percentage
All-cause mortality	10,865	2.7 (1.3)	1–11	100
Sex				
Male	6,146	1.5 (1.1)	0–10	56.6
Female	4,719	1.2 (1.0)	0–6	43.4
*Age group*				
0–4 years	2,598	0.6 (0.8)	0–5	23.9
5–19 years	3,877	0.2 (0.5)	0–4	35.7
20–59 years	972	1.0 (0.9)	0–7	8.9
60+	3,418	0.9 (0.9)	0–6	31.5

**Table 2:** Summary statistics of daily temperature and humidity in the Kintampo HDSS area from 2005 to 2015.

Variable	Mean (standard deviation)	Range	Interquartile range
Minimum temperature (°C)	23.4 (2.1)	14.0–32.0	22.0–24.6
Maximum temperature (°C)	31.0 (2.6)	22.0–39.0	29.0–33.0
Average temperature (°C) ǂ	27.2 (1.8)	20.0–34.0	26.0–28.5
AM relative humidity (%)	87.9 (14.6)	7.0–100.0	83.0–98.0
PM relative humidity (%)	65.9 (17.1)	6.0–100.0	55.0–76.0
Average relative humidity (%) ð	76.9 (14.5)	7.0–99.5	70.0–86.5
Apparent temperature (°C)	24.8 (1.6)	17.9–30.6	23.7–25.9

ǂ: average of minimum and maximum daily temperature; ð: average of maximum (AM) and minimum (PM) relative humidity.

**Table 3:** Relative risk of mortality at different apparent temperatures stratified by sex at different lag periods.

Classification	Lowest at (18°C) RR (95% CI)	First quartile at (23°C) RR (95% CI)	Third quartile at (26°C) RR (95% CI)	Highest at (31°C) RR (95% CI)
All deaths				
Lag 0 1	0.50 (0.24, 1.01)	1.03 (0.98, 1.09)	1.03 (0.99, 1.07)	0.94 (0.60, 1.47)
Lag 2–4	3.16 (1.58, 6.33)	0.93 (0.87, 0.99)	0.98 (0.93, 1.02)	1.71 (1.03, 2.83)
Lag 5–10	1.08 (0.36, 3.24)	1.01 (0.93, 1.10)	1.01 (0.95, 1.07)	0.87 (0.43, 1.79)

Male				
Lag 0-1	0.31 (0.10, 0.94)	1.03 (0.94, 1.12)	1.05 (0.99, 1.12)	1.28 (0.66, 2.46)
Lag 2–4	2.21 (0.76, 6.44)	0.93 (0.85, 1.02)	0.97 (0.91, 1.04)	2.19 (1.03, 4.65)
Lag 5–10	1.14 (0.22, 5.80)	0.99 (0.87, 1.12)	1.00 (0.91, 1.09)	1.55 (0.54, 4.50)

Female				
Lag 0-1	0.85 (0.25, 2.94)	1.04 (0.94, 1.15)	1.00 (0.93, 1.08)	0.61 (0.26, 1.42)
Lag 2–4	4.88 (1.40, 16.99)	0.93 (0.83, 1.04)	0.98 (0.90, 1.07)	1.23 (0.48, 3.15)
Lag 5–10	0.79 (0.10, 6.30)	1.05 (0.90, 1.23)	1.04 (0.93, 1.16)	0.39 (0.10, 1.50)

AT: apparent temperature; RR: relative risk; °C: degree Celsius; CI: confidence interval.

## Data Availability

The datasets used and/or analysed during the current study are available at enquiries@kintampo-hrc.org upon reasonable request.

## Abbreviations

NASA: National Aeronautics and Space Administration
AT: Apparent temperature
GSS: Ghana Statistical Service
CHPS: Community-Based Health Planning and Services
KHDSS: Kintampo Health and Demographic Surveillance System
CKI: Community Key Informant
DLNM: Distributed lag nonlinear model.
